# Renal Cell Carcinoma Is Abrogated by p53 Stabilization through Transglutaminase 2 Inhibition

**DOI:** 10.3390/cancers10110455

**Published:** 2018-11-19

**Authors:** Seon-Hyeong Lee, Won-Kyu Lee, Nayeon Kim, Joon Hee Kang, Kyung-Hee Kim, Seul-Gi Kim, Jae-Seon Lee, Soohyun Lee, Jongkook Lee, Jungnam Joo, Woo Sun Kwon, Sun Young Rha, Soo-Youl Kim

**Affiliations:** 1 Tumor Microenvironment Research Branch, Division of Cancer Biology, National Cancer Center, Goyang, Gyeonggi-do 10408, Korea; shlee1987@gmail.com (S.-H.L.); gre7th@gmail.com (W.-K.L.); nykim0117@gmail.com (N.K.); wnsl2820@gmail.com (J.H.K.); 74294@ncc.re.kr (S.-G.K.); ljs891109@gmail.com (J.-S.L.); dlalfo1234@gmail.com (S.L.); 2 New Drug Development Center, Osong Medical Innovation Foundation, Cheongju, Chungbuk 28160, Korea; 3 Department of Chemistry, College of Science, Dongguk University, 30 Pildong-ro 2-gil, Jung-gu, Seoul 04620, Korea; 4 Omics Core Lab, National Cancer Center, Goyang, Gyeonggi-do 10408, Korea; kyunghee@ncc.re.kr; 5 College of Pharmacy, Kangwon National University, Chuncheon, Gangwon-do 24341, Korea; jkl@kangwon.ac.kr; 6 Biometric Research Branch, Division of Cancer Epidemiology and Prevention, National Cancer Center, Goyang, Gyeonggi-do 10408, Korea; jooj@ncc.re.kr; 7 Songdang Institute for Cancer Research, Yonsei University College of Medicine, Seoul 03722, Korea; WOOSUN0521@yuhs.ac; 8 Songdang Institute for Cancer Research, Division of Medical Oncology, Department of Internal Medicine, Yonsei Cancer Center, Yonsei University College of Medicine, Seoul 03722, Korea; RHA7655@yuhs.ac

**Keywords:** streptonigrin, renal cell carcinoma, p53, apoptosis, transglutaminase 2

## Abstract

In general, expression of transglutaminase 2 (TGase 2) is upregulated in renal cell carcinoma (RCC), resulting in p53 instability. Previous studies show that TGase 2 binds to p53 and transports it to the autophagosome. Knockdown or inhibition of TGase 2 in RCC induces p53-mediated apoptosis. Here, we screened a chemical library for TGase 2 inhibitors and identified streptonigrin as a potential therapeutic compound for RCC. Surface plasmon resonance and mass spectroscopy were used to measure streptonigrin binding to TGase 2. Mass spectrometry analysis revealed that streptonigrin binds to the N-terminus of TGase 2 (amino acids 95–116), which is associated with inhibition of TGase 2 activity in vitro and with p53 stabilization in RCC. The anti-cancer effects of streptonigrin on RCC cell lines were demonstrated in cell proliferation and cell death assays. In addition, a single dose of streptonigrin (0.2 mg/kg) showed marked anti-tumor effects in a preclinical RCC model by stabilizing p53. Inhibition of TGase 2 using streptonigrin increased p53 stability, which resulted in p53-mediated apoptosis of RCC. Thus, targeting TGase 2 may be a new therapeutic approach to RCC.

## 1. Introduction

Renal cell carcinoma (RCC), the most common malignancy of the adult kidney, is resistant to both radiation and chemotherapy; therefore, the prognosis remains poor [1]. Although the median progression-free survival time has doubled due to first-line or second-line therapies that target tumor angiogenesis [2,3], about one-third of all patients with RCC develop metastatic disease [4]. Therefore, there is an unmet need for anti-cancer therapeutics that are effective against RCC.

Unexpectedly, we found that RCC expresses high levels of transglutaminase 2 (E.C. 2.3.2.13; TG2 or TGase 2) [5,6]. TGase 2 is responsible for the pathogenesis of inflammatory disorders, neurodegeneration, and cancers. In addition, TGase 2 plays important roles in fibroblast function [7], wound healing [8], macrophage phagocytosis [9], in the development of cancers [10,11,12], and neurological disorders such as Huntington’s and Alzheimer’s diseases [13,14,15]. Other biological functions of TGase 2 have been discussed in reviews [16,17]; these include nuclear factor κ-light-chain-enhancer of activated B cells (NF-κB) activation through inhibition of nuclear factor of κ light polypeptide gene enhancer in B-cells inhibitor α (I-κBα) [18], hypoxia-inducible factor 1-α (HIF-1α) activation through inhibition of von Hippel-Lindau tumor suppressor (VHL) [19], and suppression of cancer cell apoptosis through inhibition of p53 [5,17].

TGase 2 is regulated directly by miR-1285. Significant inhibition of RCC proliferation and invasion is induced by silencing TGase 2. Significant downregulation of miR-1285 plays a key role in the proliferation, invasion, and migration of RCC [20]. Immunohistochemistry studies show that expression of TGase 2 in RCC specimens is significantly higher than that in normal renal tissues [20,21,22]. Microarray data analysis also shows a significant increase in TGase 2 expression in RCC [6,20,23]. Previously, we reported the unexpected finding that inhibiting TGase 2 inhibits RCC tumor growth through induction of apoptosis via p53 stabilization [23]. Expression of TGase 2 is highly upregulated in the majority of clear cell RCC patient tumors and cell lines [6,22,23]. Analysis of TGase 2 microarray data revealed that expression of TGase 2 in cancer tissue is much higher than that in normal tissue [6,22,23]. Increased TGase 2 expression enables cancer cells to escape cell death by depleting p53 via TGase-2-mediated autophagy [5,6]. TGase 2 is designated as a protein-glutamine γ-glutamyltransferase (E.C. 2.3.2.13); this is based on a catalytic mechanism whereby the primary amide group of the p53-glutamine substrate is converted into a secondary amide group of a polyamine, or into an ε-amide group of p53-lysine through an iso-peptide bond [24]. This intra- or inter-molecular cross-linking results in an aggregated protein, which is degraded rapidly [25]. Recently, we found that the N-terminus of p62 [amino acids (aa) 85–110] interacts directly with the C-terminus of TGase 2 (aa 592–687), while the N-terminus of p53 (aa 15–26) interacts with the N-terminus of TGase 2 (aa 1–139) [6]. Therefore, the development of a TGase 2 inhibitor may be a useful therapeutic approach for RCC. Here, we identify a TGase 2 inhibitor, streptonigrin, which binds to the N-terminus of TGase 2 and may be a useful therapeutic for RCC.

## 2. Results

### 2.1. Expression of TGase 2 Is Increased in RCC Clinical Samples and Is Associated with Survival of RCC Patients

**Figure 1:**
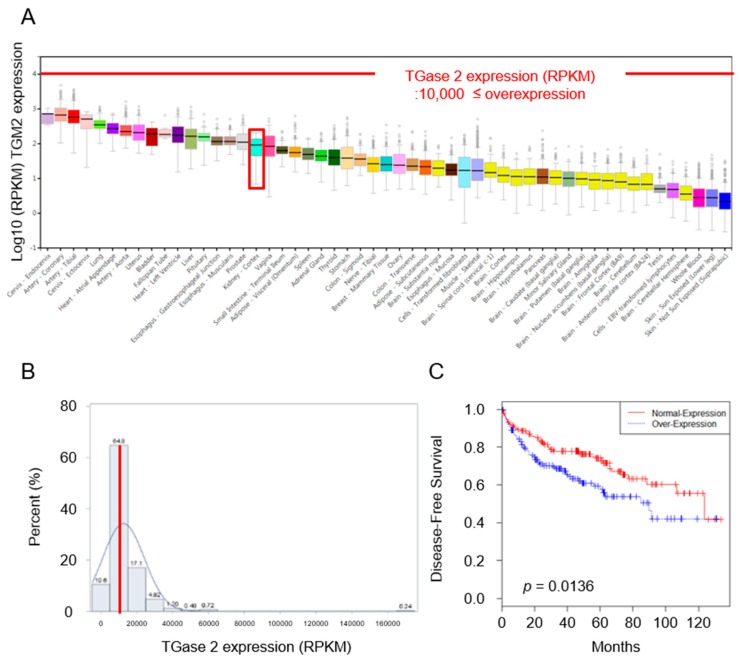
Targeting TGase 2 as a therapeutic approach to renal cell carcinoma (RCC). (**A**) TGase 2 expression in normal tissue (data from The Genotype-Tissue Expression (GTEx) Project). The final pilot analysis data set comprised 1641 samples from across 43 tissues and 175 donors. This included 18 samples from four surgical donors (SSA3, TMZS, VUSH, and WCDI) and 1623 samples from 171 postmortem donors. TGase 2 expression in 43 various normal tissues (assessed by RNA sequencing and analyzed in terms of RPKM) revealed that normal renal tissue (*n* = 32) ranked 17th (median value, 91.88 (log10 = 1.963)). (**B**) TGase 2 expression in renal cancer tissues. TGase 2 expression in 415 renal cancer patients was variable. Based on the highest expression in normal tissue (10,000 RPKM), subjects were categorized into a normal expression group (*n* = 219) if the expression level was <10,000 (mean ± SD: 6746.9 ± 2198.2) and over-expression group (*n* = 196) if the level was >10,000 (mean ± SD: 19,089.9 ± 14,248.1). (**C**) Kaplan–Meier survival curves based on TGase 2 expression. Disease-free survival (DFS) was shorter in the TGase 2 over-expressing group (*p* = 0.0136).

Genome information derived from healthy individuals was obtained from The Genotype-Tissue Expression (GTEx) project. *TGM2* expression levels and clinical information about kidney cancer patients were obtained from cBioPortal. We confirmed the expression of *TGM2* in 43 normal tissues through RNA sequencing. In terms of reads per kilobase million (RPKM), normal renal tissue (*n* = 32 samples) ranked 17th in terms of *TGM2* expression (based on median values), see Figure 1A. *TGM2* expression in renal cancer tissues was divided into two groups, which were then analyzed against the normal tissue with the highest value (Artery-coronary: ~10,000 RPKM). Clinical data regarding *TGM2* expression in 415 RCC patients from The Cancer Genome Atlas and clinical information (age, sex, and survival status) were analyzed. *TGM2* expression in 415 RCC patients ranged from 874.8–169,970.5 RPKM (mean ± SD: 12,576.4 ± 11,671.6), see Figure 1B. Based on the highest expression in normal tissue (10,000 RPKM), subjects were categorized as follows: a normal expression group (*n* = 219), which had expression levels <10,000 (mean ± SD: 6746.9 ± 2198.2) and an over-expression group (*n* = 196), which had expression levels >10,000 (mean ± SD: 19,089.9 ± 14,248.1), see Figure 1B.

Kaplan–Meier survival analysis based on TGase 2 expression revealed that disease-free survival (DFS) in the *TGM2* over-expressing group was shorter than that in the normal expression group (89.8 months vs. 123.7 months, respectively; *p* = 0.0136), see Figure 1C. Overall, 47.2% of renal cancers overexpressed *TGM2*, which is very high. 

### 2.2. Streptonigrin Inhibits TGase 2 by Interacting with the β-Sandwich Domain

**Figure 2:**
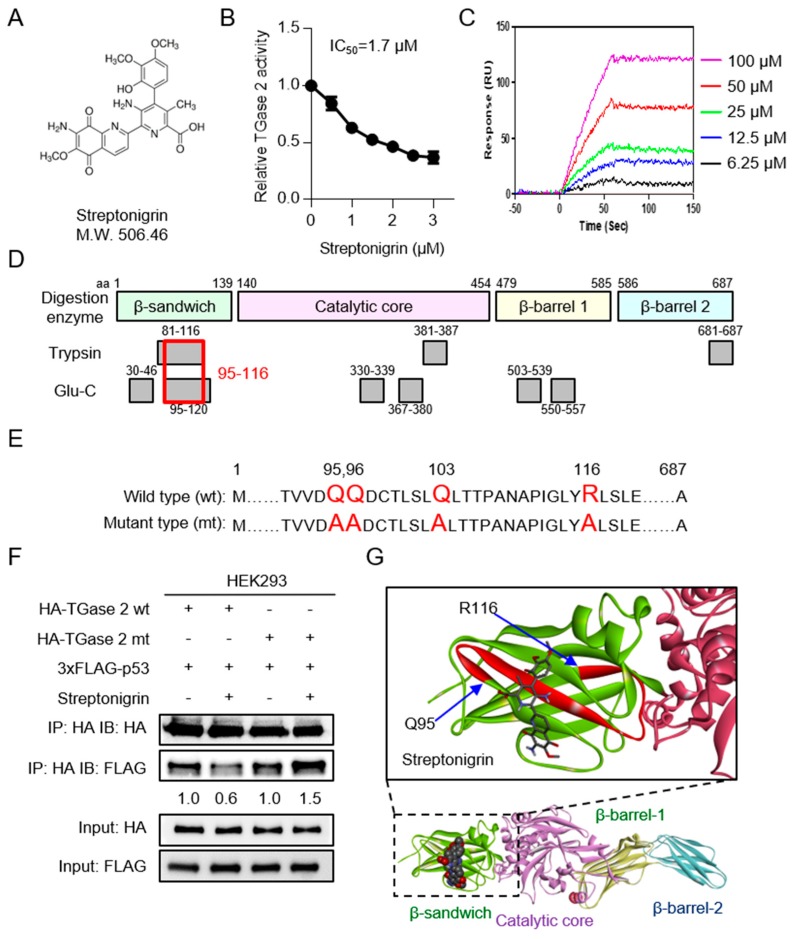
Identification and validation of streptonigrin as a TGase 2 inhibitor. In RCC, streptonigrin competes with p53 for binding to the N-terminus of TGase 2. (**A**) Chemical structure of streptonigrin. (**B**) The IC_50_ value of streptonigrin against TGase 2 is 1.7 μM. For analysis, guinea pig liver TGase 2, succinylated casein, and ^14^C-putrescine were used as competitors of streptonigrin. (**C**) SPR analysis of the interaction between streptonigrin and TGase 2. Sensograms showing binding of streptonigrin to histidine-tagged TGase 2 proteins immobilized on the tris-NTA poly ProteOnTM HTE sensor chip (binding was measured using the ProteOnTM XPR36 biosensor). The dissociation constant was calculated from association and dissociation sensograms obtained using 6.25 (black), 12.5 (blue), 25 (green), 50 (red), and 100 (purple) μM streptonigrin. The Kd value was calculated as 0.7 μM. (**D**) Summary of peptide-matching analysis. To identify the binding site of streptonigrin on TGase 2, TGase 2 and streptonigrin were incubated for 30 min at RT, followed by mass spectrometry (MS) analysis. The mass coverage of the samples revealed a difference between TGase 2 alone and the combination of TGase 2 plus streptonigrin. The streptonigrin binding-mediated masking region of TGase 2 is denoted by underlining (aa 81–116, 381–387, and 681–687 after digestion in trypsin solution, and aa 30–46, 95–120, 330–339, 367–380, 503–539, and 550–557 after digestion with Glu-C). The merged streptonigrin binding-mediated masking region of TGase 2 after trypsin and Glu-C digestion covers aa 95–116. (**E**) To test whether streptonigrin competes with p53 for binding to the N-terminus of TGase 2 (aa 95–116) through a charged interaction, we constructed a HA-pcDNA3.1/TGase 2 quadruple-point mutant-type plasmid (Q95A, Q96A, Q103A, R116A). The p3xFLAG p53 plasmid was constructed based on the p3xFLAG vector. (**F**) HEK293 (human embryonic kidney 293) cells were co-transfected with wild-type or mutant HA-TGase 2 plus the FLAG-p53 plasmids, treated with streptonigrin (100 nM) for 24 h and harvested prior to analysis of binding between TGase 2 and p53 by immunoprecipitation (IP) and immunoblotting (IB). IP of TGase 2 was performed using an anti-HA antibody and IB of p53 was performed using an anti-FLAG antibody. Streptonigrin-mediated inhibition of TGase 2 binding to p53 was analyzed by densitometry. (**G**) A complex model of TGase 2 bound to streptonigrin. The overall structural domain of TGase 2 and the streptonigrin docking site are shown. Streptonigrin docks to the β-sandwich domain of TGase 2. The expanded solid box shows the region of TGase 2 that harbors the streptonigrin binding site (aa 95–116). Streptonigrin is shown within the binding site (black stick backbone). The red loop region denotes the amino acids participating in the binding of streptonigrin (identified by MS analysis). The docking models were prepared using Discovery studio ver. 4.5.

To develop an anti-cancer drug that targets TGase 2, we screened the National Cancer Institute compound library. We selected NSC45383 (streptonigrin; CAS registration number 3930-19-6), which is a metal-dependent quinone-containing antibiotic produced by *Streptomyces flocculus*, see Figure 2A. Next, we used a casein-putrescine-based assay to test the inhibitory effects of streptonigrin on the activity of TGase 2. Streptonigrin inhibited TGase activity with an IC_50_ (half maximal inhibitory concentration) of 1.7 μM, as shown in Figure 2B. To investigate the interaction between streptonigrin and TGase 2, we performed kinetic analysis using surface plasmon resonance (SPR). Application of streptonigrin to polyhistidine-tagged TGase 2 immobilized on the nitrilotriacetic acid-functionalized SPR sensor chip resulted in dose-dependent binding, see Figure S2C. The apparent dissociation constant between streptonigrin and TGase 2 was about 0.7 μM. These results indicate that streptonigrin binds to TGase 2.

Next, we used mass spectrometry (MS) to identify the streptonigrin binding region in TGase 2. First, we established conditions that encompassed almost all TGase 2 sequences; we then compared the masses of peptides derived from TGase 2 with those derived from TGase 2 bound to streptonigrin, see Figure 2D. In contrast to TGase 2 alone, the combination of TGase 2 and streptonigrin failed to yield a peptide mass in the 95–116 aa range, see Figure S1A,E,F and Figure S1B,G,H; solid black box, suggesting that this region binds strongly to streptonigrin. We used cystamine as a negative control because it inhibits TGase 2 activity through oxidation of the disulfide bond [26]. We used MS to identify the cystamine-binding region within TGase 2. In contrast to TGase 2 alone, the combination of TGase 2 plus cystamine failed to yield a peptide mass in the aa 381–387, 419–425, or 503–523 range, see Figure S1C,D. To test whether streptonigrin competes with p53 for binding to the N-terminus (aa 95–116) of TGase 2 through a charged interaction, we constructed the HA-pcDNA3.1/TGase 2 quadruple-point mutant-type plasmid (Q95A, Q96A, Q103A, R116A), see Figure 2E. Human embryonic kidney 293 (HEK293) cells were co-transfected with wild-type or mutant HA-TGase 2 plus FLAG-p53 plasmids, treated with streptonigrin and then harvested for analysis of binding between TGase 2 and p53 by immunoprecipitation (IP) and immunoblotting (IB), see Figure 2F. The mutant form of TGase 2 failed to decrease p53 binding upon streptonigrin treatment; by contrast, wild-type TGase 2 decreased p53 binding by 40% upon streptonigrin treatment, see Figure 2F. Streptonigrin-mediated inhibition of TGase 2 binding to p53 was analyzed by densitometry. This region is remote from the cysteine triad of C370, C371, and C230 [27,28] in addition to the active site C277. The docking model for TGase 2 and streptonigrin was obtained using Discovery studio ver. 4.5. TGase 2 contains a binding pocket for streptonigrin at aa 95–116, see Figure 2G. 

To test whether streptonigrin binding to TGase 2 is dependent on its unfolded form, we analyzed native gels run after TGase 2 was incubated with streptonigrin, see Figure S2. TGase 2 is stable in an unfolded monomeric form at 20 °C but adopts an unfolded dimeric form (through binding of the C-termini) at 37 °C [29]. The unfolded monomeric form of TGase 2 was converted to the folded form by treatment with guanosine triphosphate (GTP), or to an unfolded dimer at 37 °C, see Figure S2. IB analysis of TGase 2 revealed a 2.4-fold increase in the density of the dimer band after streptonigrin treatment, see Figure S2, which suggests that binding of streptonigrin to TGase 2 may induce a conformational change that accelerates TGase 2 dimerization, see Figure S2. A previous study revealed that the PB1 domain of p62 (residues 85–110) interacts with the C-terminal domain of TGase 2 (residues 592–687) [6]. The N-terminus of TGase 2 binds to p53 and simultaneously associates with p62 through its C-terminus [6]. The LC3 binding domain of p62 is located within its C-terminus, which facilitates the formation of autophagosomes [6]. Therefore, binding of streptonigrin to the N-terminus of TGase 2 increases the stability of p53.

### 2.3. Streptonigrin Stabilizes p53 by Inhibiting TGase 2

**Figure 3:**
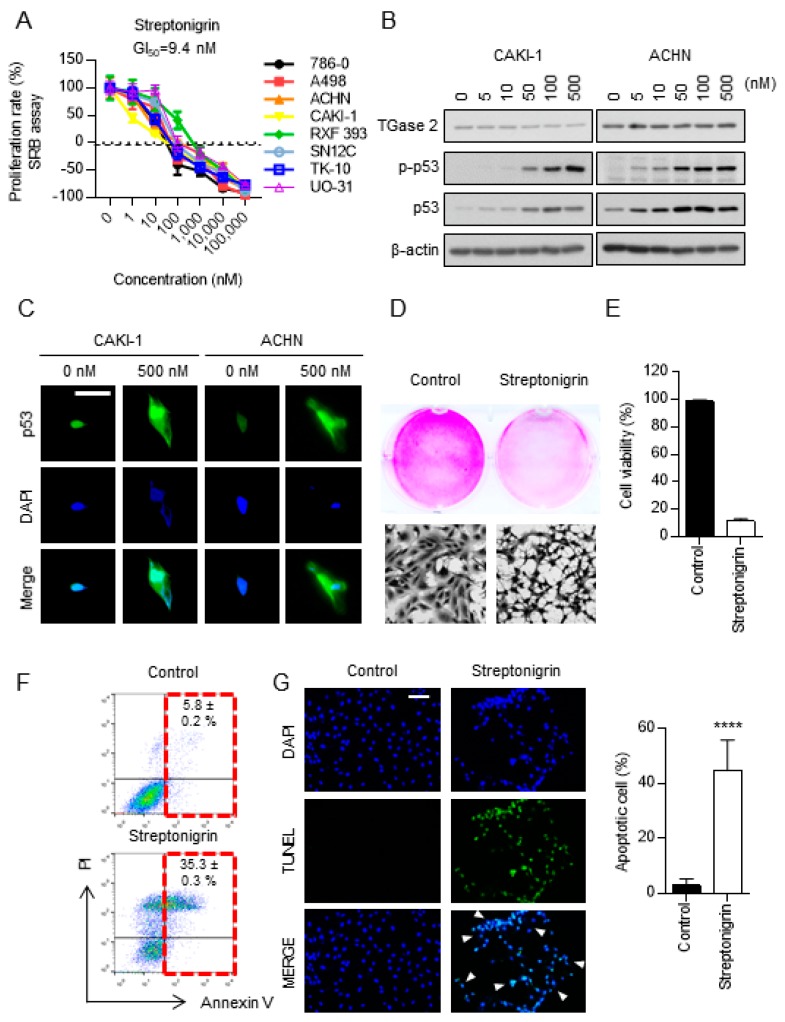
Streptonigrin induces apoptosis of RCC cells by stablizing p53 via TGase 2 inhibition. (**A**) The anti-proliferative activity of streptonigrin was evaluated in a sulforhodamine B (SRB) assay with a panel of human RCC cells. Cells were treated for 48 h with the indicated concentrations of streptonigrin. The GI_50_ of streptonigrin was 9.4 nM. (**B**) Streptonigrin treatment (for 4 h) of CAKI-1 and ACHN cells stabilized phospho-p53 and p53 in a dose-dependent manner. TGase 2 expression was unchanged. (**C**) Immunofluorescence staining of p53 (green) in CAKI-1 and ACHN cells treated with 500 nM streptonigrin for 4 h. Images show stable expression of p53 in the presence of streptonigrin. Nuclei were stained with 4′,6-diamidino-2-phenylindole (DAPI) (blue). Scale bar = 50 μm. (**D**) Streptonigrin suppresses proliferation of CAKI-1 cells. Cells were treated for 6 h with streptonigrin (0 or 500 nM), fixed in 4% paraformaldehyde, and stained with sulforhodamine B (SRB) (original magnification, 10×). (**E**) Viability of CAKI-1 cells treated (or not) with streptonigrin for 6 h (500 nM, *n* = 3) was measured in a trypan blue exclusion assay. (**F**) Cells were treated for 6 h with streptonigrin (0 or 500 nM) and stained with propidium iodide and annexin V prior to analysis by flow cytometry. LL (**lower left**), living cells; UL (**upper left**), necrotic cells; LR (**lower right**), apoptotic cells; UR (**upper right**), dead cells (*n* = 3). (**G**) Cells were treated for 6 h with streptonigrin (0 or 500 nM) and then subjected to a TUNEL assay to detect apoptosis. The bar graph shows the percentage (mean ± SD) of apoptotic cells in at least five randomly selected fields of view (****, *p* < 0.0001). Scale bar = 100 μm.

Streptonigrin showed potent, dose-dependent inhibitory effects on RCC growth, as shown in Figure 3A and Table S3. Furthermore, the GI_50_ (the concentration that inhibits growth by 50%) of streptonigrin suggested that it is more cytotoxic than sunitinib or sorafenib, see Figure S3A and Table S3. Furthermore, the GI_50_ of streptonigrin against RCC cell lines (9.4 nM) showed that it is more toxic to these cells than to human umbilical vein endothelial cells (GI_50_~117.5 nM) and mouse embryonic fibroblast cells (~24.7 nM), see Figure S3B,C. Treatment of ACHN and CAKI-1 cell lines with streptonigrin led to a dose-dependent increase in the amount of p53; however, the amount of TGase 2 remained constant, see Figure 3B. Immunofluorescence staining confirmed the increase in p53 expression by CAKI-1 and ACHN, see Figure 3C. Sulforhodamine B (SRB) and trypan blue exclusion assays showed that streptonigrin decreased the number of viable cells by ~90%, see Figure 3D,E. In addition, flow cytometry and terminal deoxynucleotidyl transferase dUTP nick-end labeling (TUNEL) staining revealed an ~6-fold increase in cell death, as shown in Figure 3F,G. To test whether streptonigrin affects serum proteins, we measured the concentrations of p53 and TGase 2 in fetal bovine serum (FBS; 0%, 5%, and 10%) for 24 h, followed by addition (or not) of streptonigrin (0.1 μM) for 4 h, see Figure S3D. Serum concentration had no effect on the streptonigrin-mediated stabilization of p53, see Figure S3D.

**Figure 4:**
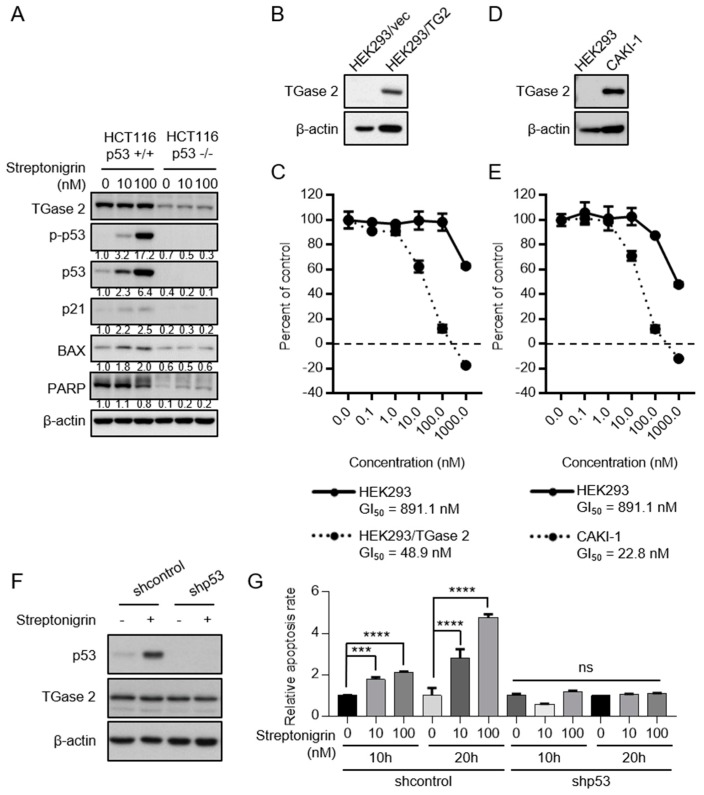
Streptonigrin acts as a specific TGase 2 inhibitor and induces p53-dependent cell death in HCT116 and HEK293 cells. (**A**) HCT116(p53 +/+) and HCT116(p53 −/−) cells were treated for 12 h with different doses of streptonigrin and subjected to immunoblotting with the indicated antibodies. (**B**) Western blot showing TGase 2 expression in HEK293 and HEK293/TGase 2 cells (transiently transfected with TGase harboring a 2-HA tag). (**C**) Sulforhodamine B (SRB) assay to examine streptonigrin-induced cytotoxicity in HEK293 and HEK293/TGase 2 cells exposed to the indicated concentrations of the compound. (**D**) Western blot showing TGase 2 expression in HEK293 and CAKI-1 cells. (**E**) SRB assay of streptonigrin cytotoxicity in HEK293 and CAKI-1 cells exposed to the indicated concentrations of the compound. (**F**) CAKI-1 shcontrol and shp53 cells were treated with streptonigrin (100 nM) for 10 h and analyzed by immunoblotting with the indicated antibodies. (**G**) Cells were treated for 10 h or 20 h with streptonigrin (0, 10, or 100 nM) and stained with propidium iodide and annexin V and analyzed by FACS flow cytometry. ns > 0.05; *** *p* < 0.001 and **** *p* < 0.0001.

HCT116(p53 +/+) or HCT116(p53 −/−) cell lines were treated with streptonigrin to test whether streptonigrin-induced apoptosis depends on p53 stabilization. Streptonigrin (doses of 10 nM and above) increased expression of p53, phospho(p)-p53, p21, and BAX in HCT116(p53 +/+) cells in a dose-dependent manner, as shown in Figure 4A. However, treatment of HCT116(p53 −/−) cells did not activate p21, BAX, or poly (Adenosine diphosphate-ribose, ADP-ribose) polymerase (PARP), see Figure 4A. Furthermore, a proliferation assay using cells expressing high amounts (HEK293/TGase 2 or CAKI-1) or low amounts (HEK293) of TGase 2 showed that streptonigrin was more cytotoxic to the former, as shown in Figure 4B–E. At a concentration of 100 nM, streptonigrin killed 80% of HEK293/TGase 2 cells but only 10% of HEK293 cells, see Figure 4B,C. At the same concentration, streptonigrin killed 90% of CAKI-1 cells but only 10% of HEK293 cells, see Figure 4D,E. Taken together, these results suggest that streptonigrin induces the death of cells expressing high amounts of TGase 2 by stabilizing p53 via inhibition of TGase 2, see Figure 4C,E. The results are in agreement with those of previous reports showing that TGase 2 knockdown [5] or inhibition with GK921 [23] induces cell death by stabilizing p53. To confirm that streptonigrin-induced death of RCC cells depends on p53, p53 was knocked down and cells were treated with streptonigrin, see Figure 4F,G. P53-knockdown RCC cells did not undergo apoptosis in the presence of streptonigrin, whereas RCC cells harboring wild-type p53 showed a 2-fold increase in apoptosis after exposure to streptonigrin, as shown in Figure 4G.

### 2.4. A Single Dose of Streptonigrin Inhibits the Growth of RCC Xenografts

**Figure 5:**
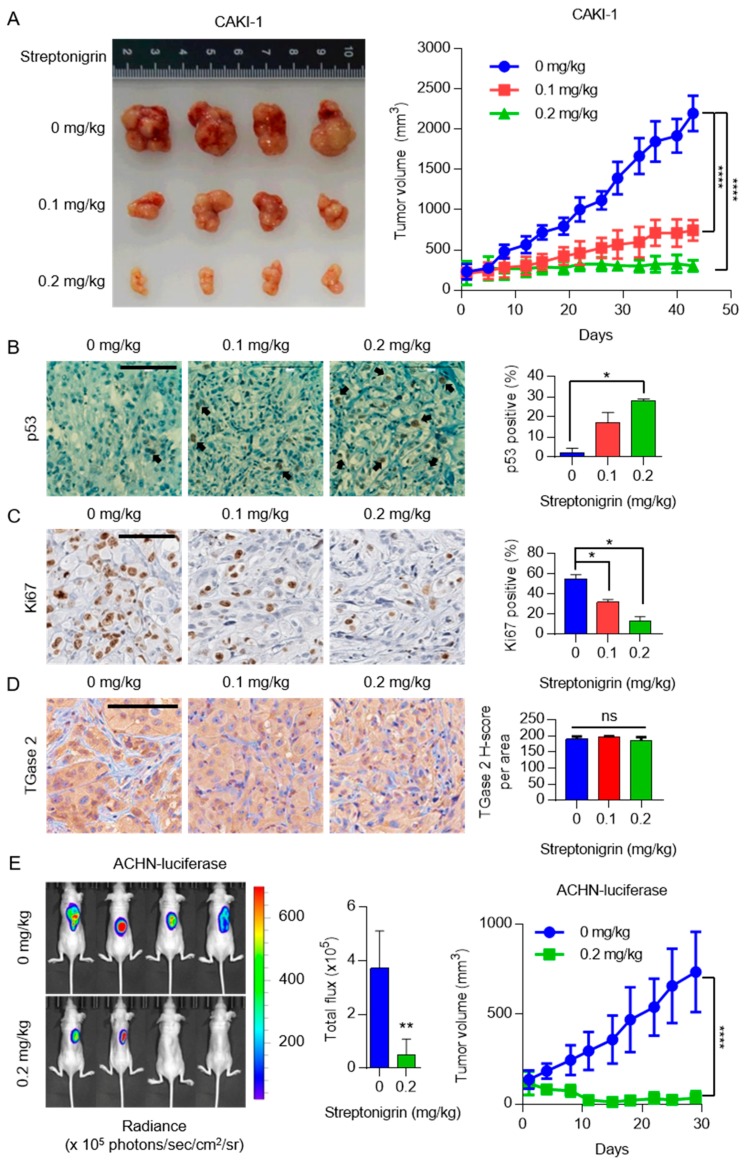
Streptonigrin shows anti-cancer activity in a human CAKI-1 xenograft model. (**A**) CAKI-1 cells were injected subcutaneously into one flank of BALB/c nude mice (*n* = 4). Streptonigrin treatment was initiated when tumors reached a volume of 250 mm^3^. Photograph of tumors that developed in each group. The average volume of tumors in BALB/c nude mice is represented as the mean ± SD. (**B**–**D**) CAKI-1 tumors harvested from BALB/c nude mice were analyzed by immunohistochemistry using antibodies specific for (**B**) p53, (**C**) Ki67, and (**D**) TGase 2 (100× magnification). Scale bar = 100 μm. The bar graph represents the percentage of cells positive for p53 and Ki67. Data are expressed as the mean ± SD (*n* = 3). (**E**) Luciferase-tagged ACHN cells were injected subcutaneously into one flank of BALB/c nude mice. Streptonigrin treatment was initiated when tumors reached a volume of 130 mm^3^. Each treatment group comprised four mice treated with vehicle (control), or with 0.2 mg/kg streptonigrin, for 29 days. Tumor growth was measured and calculated as described in the methods. The bar graph represents the Xenogen imaging signal intensity (photons/sec/cm^2^/steradian) at 30 days (mean ± SD, *n* = 4). ns *p* > 0.05, * *p* < 0.05; ** *p* < 0.01, and **** *p* < 0.0001.

**Figure 6:**
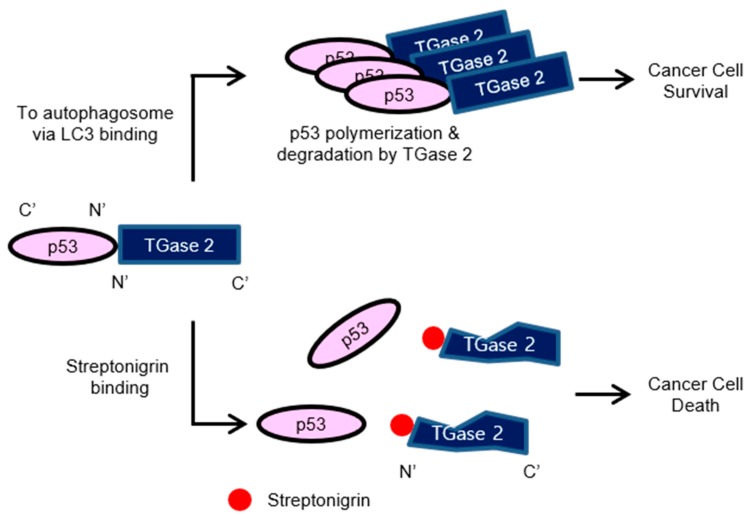
Summary of the role of TGase 2 in RCC. Inhibition of TGase 2 by streptonigrin triggers cell death by stabilizing p53.

Given the above in vitro results, we next tested whether streptonigrin has a therapeutic effect in a human RCC xenograft model. For this, we used two different xenograft models to analyze tumor growth: an invasive tumor growth model with CAKI-1 cells and a non-invasive tumor model with luciferase-tagged ACHN cells. A single treatment with streptonigrin reduced CAKI-1 tumor growth in a dose-dependent manner over six weeks, as shown in Figure 5A. After 43 days, tumors in streptonigrin-treated mice were approximately 10-times smaller than those in control mice, see Figure 5A. Tumors were collected at the end of the study and immunohistochemical staining for TGase 2, p53, and Ki67 was performed, see Figure 5B–D. The results showed a robust expression of p53 in tumors from streptonigrin-treated mice, whereas p53 positive staining was almost absent from the controls, see Figure 5B. Ki67 staining showed a clear inverse correlation with streptonigrin treatment, see Figure 5C. TGase 2 expression was unchanged by streptonigrin treatment, as shown in Figure 5D. Imaging of non-invasive luciferase-expressing ACHN tumors was conducted by measuring bioluminescence after luciferin injection using the Xenogen instrument. After 29 days of treatment, tumor volume in the streptonigrin-treated group was approximately 10-times lower than that in the controls, see Figure 5E. Analysis of Xenogen images revealed that the volume of tumors in the streptonigrin-treated group was approximately 7-times smaller than that in the controls, see Figure 5E. No mice died or experienced body weight changes after treatment with 0.2 mg/kg streptonigrin for six weeks, see Figure S4A,B. To summarize, TGase 2 promotes autophagy-dependent degradation of p53 in RCC cell lines, which increases RCC cell survival, see Figure 6. However, streptonigrin prevents p53 depletion by inhibiting TGase 2, which induces cell death by increasing the stability of p53, see Figure 6.

## 3. Discussion

Here, we focused on the anti-cancer effects of the TGase 2 inhibitor streptonigrin, which was selected by screening a drug library. In addition, we showed that these effects were mediated via streptonigrin-induced apoptosis of RCC cells triggered by stabilization of p53, which concurs with our previous report [5]. We have demonstrated multiple times that the TGase 2 inhibitor GK921 [6,23] or streptonigrin (this paper) induces a marked remission of RCC tumors in models based on RCC cell lines such as ACHN and CAKI-1. The most important finding of the present work is that the induction of tumor remission after TGase 2 inhibition is associated with increased stability of p53. Recently, we showed that GK921 binds to the N-terminus of TGase 2 (aa 81–116), which stabilizes p53 by blocking TGase 2 binding [30,31]. Binding of GK921 to the N-terminus of TGase 2 also deactivates TGase 2 through non-covalent self-polymerization of TGase 2, which is induced via a conformational change [31]. TGase 2 contains an extracellular trafficking sequence at the N-terminus (aa 88–106) of the β-sandwich domain [32]. Here, we showed that streptonigrin also binds to the N-terminus of TGase 2 (aa 95–116) and inhibits TGase 2 activity, although it does not bind to the active site of TGase 2. This suggests that the N-terminus is an important feature of TGase 2. 

We built a comprehensive model of the streptonigrin/TGase2 complex based on MS results and docking analysis using the small angle X-ray scattering/molecular dynamics (SAXS/MD) dimer model [29]. Although we could not fully determine the X-ray crystallography structure of the TGase 2–streptonigrin complex, we were able to deduce a potential inhibitory mechanism based on experimentally supported results, such as inhibitory kinetics against TGase 2, see Figure 2B, SPR-binding of streptonigrin to TGase 2, see Figure 2C, and MS analysis after binding of streptonigrin to TGase 2, see Figure 2D and Figure S1A,B. This suggested mechanism is consistent, see Figure 2F, with the results of immunoprecipitation of streptonigrin-treated HEK293 cells transfected with TGase 2 and p53. To analyze the effect of streptonigrin on the conformation of the TGase 2 active site, we identified the catalytic triad of TGase 2 (e.g., Cys277, His335, and Asp358 residues) in the folded (1KV3) [33] and unfolded (2Q3Z) TGase 2 models [34]. Streptonigrin was not able to bind the folded form of TGase 2 [33]; however, it did bind to the unfolded form, see Figure S2.

Streptonigrin (also known as bruneomycin or rufocromomycin) is an aminoquinone anti-neoplastic antibiotic isolated from the bacterium *Streptomyces flocculus*. Streptonigrin shows structure-related activity against peptidyl-arginine deiminase (PAD), resulting in inactivation of the latter [35,36], which may result in anti-neoplastic effects. A previous study revealed that the 7-amino-quinoline-5,8-dione core of streptonigrin is a highly potent pharmacophore that acts as a pan-PAD inhibitor [36]. However, the anti-cancer effects of streptonigrin are based on inhibition of TGase 2 rather than on inhibition of PAD because the PAD inhibitor Cl-amidine does not have anti-cancer effects on RCC cell lines, see Figure S5. Streptonigrin is also thought to induce direct DNA damage via formation of a streptonigrin-metal-DNA complex. The mechanism underlying streptonigrin-mediated inhibition of DNA and RNA is thought to be based on inhibition of topoisomerase II via DNA strand breakage following reduction with NADH [37]. It was also suggested that streptonigrin cleaves DNA in vitro by forming a complex with metal ions; also, auto-oxidation of streptonigrin in the presence of NADH generates free radicals [37]. However, in vitro experimental conditions involved the use of streptonigrin at 100 μM, along with 100 μM copper II and 1 mM NADPH [37,38]. The proposed mechanism underlying the anti-cancer effects of streptonigrin suggests that its activity against RCC is likely due to TGase 2 inhibition. A previous study reported a clinical trial of streptonigrin in a small group of patients with advanced cancers [39]. Although two out of twenty-one patients attained temporary remission and one patient achieved prolonged remission, the remaining 18 showed no response. However, most cancer cases were non-RCC (i.e., leukemia, breast cancer, and brain cancer). The report also highlighted a high incidence of side effects such as nausea, thrombocytopenia, and lymphopenia; it is worth noting here that we did not observe any noticeable weight loss or behavioral problems in our xenograft models under our experimental conditions, see Figure S4. 

Although some biomarkers of RCC have been suggested [40], no RCC-specific therapeutic target has been identified. The most common therapeutics are anti-angiogenic and include pazopanib [41] and sorafenib (which target vascular endothelial growth factor receptor (VEGFR)) [42] or anti-anabolic (e.g., rapamycin [43], which targets mTOR). However, RCC is resistant to radiation, chemotherapy, and targeted therapy, which results in a poor prognosis. Therefore, our finding that expression of TGase 2 shows an inverse correlation with the expression of p53, with only a 4% mutation rate in RCC, is notable [6]. A series of studies of TGase-2-mediated p53 instability in RCC revealed that TGase 2 binds simultaneously to p53 and p62 and transfers p53 to the autophagosome through binding of p62 to LC3 [5,6,23,44]. SiRNA-mediated knockdown of TGase 2 in RCC stabilizes p53, which triggers apoptosis. It is also possible that inhibiting TGase 2 using streptonigrin may attenuate tumor growth by down-regulating NF-κB. We reported that TGase 2 activates NF-κB by depleting I-κBα through polymerization [18,45,46]. However, the contribution of TGase 2 to NF-κB activity in RCC remains unclear.

With respect to side effects, TGase 2 knockout mice show no lethality or changes in the normal phenotype [47,48]. Therefore, a TGase 2 inhibitor may be a safe and effective RCC therapeutic. Here, we showed that streptonigrin has a very strong anti-RCC effect in vivo by inhibiting TGase 2 at very low concentrations (0.2 mg/kg); thus inhibition of TGase2 may be a novel and effective therapeutic approach.

## 4. Materials and Methods

### 4.1. Antibodies and Reagents

The anti-TGase 2 (Cat. MA5-12739, 1:1000) was purchased from Thermo Scientific (Waltham, MA, USA). Antibodies specific for β-actin (Cat. #sc-47778, 1:500), p53 (Cat. #sc-126; 1:500), p21 (Cat. #sc-6246; 1:500), and BAX (Cat. #sc-20067; 1:500), and the HA-probe (human influenza hemagglutinin-probe, Cat. #sc-805; 1:500), were purchased from Santa Cruz Biotechnology (Dallas, TX, USA). The anti-FLAG antibody (peptide DYKDDDK epitope, Cat. #F1804; 1:2500) was purchased from Sigma-Aldrich (St. Louis, MO, USA). Antibodies specific for p-p53 (Cat. #9284; 1:1000) and PARP (Cat. #9542; 1:1000) were purchased from Cell Signaling Technology (Danvers, MA, USA). Streptonigrin (Cat. #S1014) and sunitinib (Cat. #PZ0012) were purchased from Sigma-Aldrich (St. Louis, MO, USA). Sorafenib (Cat. #S1040) was purchased from Selleckchem (Houston, TX, USA) and Cl-amidine (Cat. #10599) was purchased from Cayman Chemical Company (Ann Arbor, MI, USA).

### 4.2. Cell Culture

Renal cell carcinoma cell lines (786-0, A498, ACHN, CAKI-1, RXF 393, SN12C, TK-10, and UO-31) were obtained from the National Cancer Institute (MTA Number: 2702-09). Cells were cultured in complete RPMI 1640 medium (Hyclone, Logan, UT, USA) containing 10% FBS (Hyclone, Logan, UT, USA) in an atmosphere of 5% CO_2_/100% humidity at 37°C. ACHN-luc2 cells were purchased from Perkin Elmer (Waltham, MA, USA) and cultured in MEM/EBSS (Hyclone, Logan, UT, USA) containing 10% FBS. Human embryonic kidney (HEK) 293 cells were cultured in Dulbecco’s Modified Eagle’s Medium (Hyclone, Logan, UT, USA) containing 10% F. All cell lines were subjected to short-tandem repeat profiling, see Table S1.

### 4.3. Immunofluorescence Analysis

Cells were grown on coverslips, fixed in 4% paraformaldehyde for 10 min at room temperature, and washed twice with PBS (phosphate-buffered saline). Cells were then permeabilized with 0.5% Triton-X 100 for 10 min at room temperature and washed twice with PBS. Cells were blocked in 3% bovine serum albumin (BSA) for 1 h at room temperature, stained with anti-p53 antibody for 16 h, washed twice with PBS, and stained with an Alexa-488-(green)-conjugated secondary antibody. Nuclei were stained with 4′,6-diamidino-2-phenylindole (DAPI) (blue). Cells stained with the secondary antibody alone were used as a control. Cells were viewed under an Axiovert 200M laser scanning confocal microscope (Carl Zeiss, Oberkochen, Germany).

### 4.4. TGase 2 Activity Assay

Each assay vial contained 48.58 μL TEN buffer (50 mM Tris-Cl, 1 mM EDTA, 50 mM NaCl, pH 7.5) and 0.42 μL guinea pig TGase 2 (25 ng/μL). Then, 1 μL streptonigrin (at six different concentrations) was added to the assay vial. The assay vials were pre-incubated at RT for 5 min prior to the addition of 140 μL of 2% succinylated casein [5 mM dithiothreitol (DTT)] and 10 μL of 2 μCi/mL putrescine. The vials were incubated in a 37 °C thermomixer for 15 min. The reaction was stopped by adding 2 mL of cold 5% trichloroacetic acid (TCA). The assay mix was then filtered onto a glass-fiber filter paper disc (Whatman GF/A, Maidstone, UK) and washed with cold 5% TCA. The filter was placed into a counting vial and 4 mL of scintillation cocktail solution was added. The counting vial was vortexed for 5 s and placed on a shaker for 30 min before counting. The IC_50_ was calculated as the average concentration leading to 50% inhibition of enzyme activity under a specific in vitro condition. Therefore, the IC_50_ value was affected mainly by the initial amount of enzyme in the assay.

### 4.5. Construction of the TGase 2 Mutant

Construction of the TGase 2/HA-pcDNA3.1 quadruple-point mutant-type plasmid (Q95A, Q96A, Q103A, R116A) and the wild-type p53/p3xFLAG-CMV plasmid was based on wild-type plasmids that were previously described [5]. The primers are listed in Table S2. All constructs were confirmed by DNA sequencing.

### 4.6. MS Analysis of the Streptonigrin Binding Site in TGase 2

To prepare the samples, 1 mM streptonigrin was incubated with 0.5 milliunits of recombinant human TGase 2 in a reaction mixture containing 100 mM Tris, pH 7.5, 100 mM sodium chloride (NaCl), 1 mM ethylenediaminetetraacetic acid (EDTA), and 2 mM magnesium chloride (MgCl_2_) with or without streptonigrin. After 1 h at 37 °C, the reactants were analyzed by liquid chromatography–mass spectrometry (LC-MS/MS) according to a published method [31].

### 4.7. Measurement of the Interaction between Streptonigrin and TGase 2

SPR analysis of the interaction between streptonigrin and recombinant human TGase 2 was performed using the ProteOn XPR36 array system (Bio-Rad, Hercules, CA, USA). First, TGase 2 was immobilized on the ProteOn HTG sensor chip (Bio-Rad, Hercules, CA, USA) for the capture of poly-His-tag proteins according to the protocol described in the instruction manual. Sensorgrams for all binding interactions were recorded in real time and analyzed after subtracting data from the control channel. After each measurement, the surface of the sensor chip was regenerated using 300 mM EDTA or 0.5 M NaCl. The dissociation and rate constants were calculated by the ProteOn XPR36 Manager program (Bio-Rad, Hercules, CA, USA).

### 4.8. Molecular Docking Analysis

Molecular docking analysis was performed using Docking Server (http://www.dockingserver.com). Briefly, the following parameters were set in Docking Server: Grid parameter files were built and atom-specific affinity maps were constructed using Autogrid 4. The initial position, orientation, and torsion of the streptonigrin molecules were set according to the crystal structure of human TGase2 in the open conformation (PDB: 2q3z) and the MS analysis data, see Figure 2C. All rotatable torsions were released during docking. After each docking calculation, the root mean square deviation (RMSD) between the lowest energy docked ligand pose and the complex crystal structure ligand pose was evaluated, see Figure 2G. 

### 4.9. SRB Assay of Anti-Proliferative Activity

The SRB assay was performed as described [49]. Briefly, cells (100 μL medium containing 10,000–25,000 cells/well) were incubated in 96-well microtiter plates. After 24 h, the drug was added (100 μL) to each well and the cultures were incubated for 48 h at 37 °C. The cells were fixed in 50% TCA (50 μL per well) for 1 h at 4 °C. The liquid was removed from the plate, which was then rinsed five times with water and allowed to dry at room temperature (RT). Washed cells were stained for 10 min at RT with 0.4% SRB (100 μL per well). After staining, the plate was washed three times with 1% glacial acetic acid and dried at RT. The SRB stain was then solubilized in 10 mM Tris and absorbance were read at 515 nm. The effect of the drugs was expressed in terms of the GI50 (the concentration resulting in 50% maximal inhibition of cell proliferation), TGI (total growth inhibition), or LC50 (lethal concentration).

### 4.10. Viral Transduction

The human p53 (NM_000546 GenBank) MISSION shRNA set (five individual hairpins cloned individually into pLKO.1-puro; Sigma-Aldrich, St. Louis, MO, USA) were used to generate lentiviral particles in HEK293FT packaging cells. Sub-confluent HEK293T cells were co-transfected with 1.8 µg MISSION shRNA set, 0.6 µg pMD2.G, and 1.2 µg psPAX2 per 6-well tissue culture plate using Lipofectamine™ 2000. After 48 h, supernatants were collected, filtered, and used for p53 knock down by transduction of shRNA into CAKI-1 cell lines. Transduced cells were analyzed by immunoblotting with an anti-p53 antibody. CAKI-1 cells were transduced with MISSION non-target control transduction virus (scrambled RNA; SHC002V; Sigma-Aldrich, St. Louis, MO, USA).

### 4.11. Western Blotting

Whole cell lysates were prepared using radioimmunoprecipitation assay (RIPA) buffer (50 mM Tris-HCl, pH 8.0, with 150 mM sodium chloride, 1.0% igepal CA-630 (NP-40), 0.5% sodium deoxycholate, 0.1% sodium dodecyl sulfate, protease inhibitor cocktail, and phosphatase inhibitor cocktail). Protein assays were carried out using a Bradford protein assay (Thermo Scientific, Waltham, MA, USA) to normalize protein expression. Proteins were resolved by SDS-PAGE and transferred to polyvinylidene difluoride (PVDF) membranes (Merck Millipore, Burlington, MA, USA). Membranes were blocked in 5% BSA for 1 h at RT and then incubated overnight at 4°C with the indicated antibodies. Membranes were washed for 1 h at RT in TBS-T, followed by incubation with a horseradish peroxidase-conjugated secondary antibody for 1 h at RT. Finally, membranes were washed for 1 h at RT in TBS-T and developed using enhanced chemiluminescence. 

### 4.12. Measurement of Cell Viability

Cell viability was determined in a trypan blue dye exclusion assay. Cells were cultured in 2 mL of medium in 6-well plates. After treatment with streptonigrin, cells were stained with 0.4% trypan blue (Sigma-Aldrich, St. Louis, MO, USA), and both viable and non-viable cells were counted.

### 4.13. Apoptosis Assay

Analysis of Annexin V binding was conducted using the Annexin V Apoptosis Detection Kit I (BD Biosciences, Franklin Lakes, NJ, USA) according to the manufacturer’s instructions. Cells were harvested, washed twice with cold PBS, and resuspended in 1× binding buffer at a concentration of 1 × 10^6^ cells/mL. Next, 100 μL of the cell suspension (1 × 10^5^ cells) was transferred to a 5 mL culture tube and stained with 5 μL Annexin V-FITC and 5 μL propidium iodide (PI). After incubation in the dark for 15 min at RT, 400 μL 1× binding buffer was added to each tube. Data were acquired by flow cytometry within 1 h. For each sample, 10,000 ungated events were acquired: PI (−)/Annexin V-FITC (+) cells (early apoptotic population) and PI (+)/Annexin V-FITC (+) cells (late apoptotic population) were sorted. TUNEL staining of fixed cells was performed using the In Situ Cell Death Detection Kit (Roche, Indianapolis, IN, USA), according to the manufacturer’s instructions. The slides were examined under a Zeiss Axiovert 200 M microscope (Carl Zeiss Microscopy, Jena, Germany).

### 4.14. Preclinical Xenograft Tumor Models

Six-week-old, female-specific, pathogen-free BALB/c nude mice (*n* = 12) were purchased from Central Lab (Animal Inc., Seoul, Korea). Each mouse received CAKI-1 and ACHN-luc2 cells (5.0 × 10^6^ cells/head) subcutaneously. When tumors reached an appropriate size (200–250 mm^3^ for Caki-1 and 100–150 mm^3^ for ACHN-luc2), the mice were randomized into 3 groups (*n* = 4–5) according to tumor volume and body weight: the control group was treated with vehicle only (0.04% dimethyl sulfoxide [DMSO]) in PBS; the streptonigrin-treated group received 0.1 mg/kg or 0.2 mg/kg of the compound. Vehicle and streptonigrin were administered orally once per day 5 days/week. The size of the primary tumors was measured every 3–4 days using calipers. Tumor volume was calculated using the formula: *V* = (*A* × *B*^2^)/2, where *V* is the volume (mm^3^), *A* is the long diameter, and *B* is the short diameter (mm). Mice were euthanized using 7.5% CO_2_ and tumors were harvested for immunohistochemical analysis. This study was reviewed and approved by the Institutional Animal Care and Use Committee (IACUC) of the National Cancer Center Research Institute. NCCRI is an Association for Assessment and Accreditation of Laboratory Animal Care International (AAALAC International)-accredited facility and abides by the Institute of Laboratory Animal Resources (ILAR) guide (IRB number: NCC-15-126C).

### 4.15. Automated Immunohistochemistry

Immunohistochemistry analysis was performed using a VENTANA Discovery XT automated staining instrument (Ventana Medical Systems, Tucson, AZ, USA). For animal tumor staining, see Figure 4B–D, slides were prepared from xenografts and de-paraffinized for 30 min at 75 °C using EZprep solution (Ventana Medical Systems, Tucson, AZ, USA). Epitope retrieval was accomplished in the automated stainer by exposure to CC1 solution (Ventana Medical Systems, Tucson, AZ, USA) for 64 min at 95 °C. Antibodies were titered over a range of concentrations to provide the optimum specific staining to background staining ratio. Once the titers were set, antibodies specific to TGase 2, p53, and Ki67 were transferred (with diluent) to user-fillable dispensers for use on the automated stainer. Slides were developed using the Optiview DAB detection kit (Ventana Medical Systems, Tucson, AZ, USA). Briefly, the steps were as follows: inhibitor for 8 min, linker for 8 min, multimer for 12 min, DAB/peroxide for 8 min, and copper for 4 min. Slides were then counterstained with hematoxylin II for 8 min (Ventana Medical Systems, AZ, USA). Antibody titers were determined for each antibody using positive and negative control tissues, according to the manufacturer’s instructions. Representative images from each tumor were collected using a 20× objective lens. TGase 2 expression was assessed by inForm Cell Analysis (PerkinElmer). Four cell line controls were included in each batch that spanned the range of 0 (negative, no staining) to 3+ (intense staining). TGase 2 IHC was scored categorically, according to a staining intensity scale from 0 to 3+. TGase 2 IHC H-score was computed as the sum of [1 × (% cells 1+) + 2 × (% cells 2+) + 3 × (% cells 3+)].

### 4.16. In Vivo Bioluminescence Imaging

Expression of the luciferase transgene in live animals was monitored using a Xenogen IVIS Lumina imaging system (Perkin Elmer/Caliper Life Sciences, Hopkinton, MA, USA). BALB/c nude mice were injected intraperitoneally with 75 mg/kg D-luciferin (Gold Biotechnology, Olivette, MO, USA). After 3 min, anesthetized mice were imaged for 1 s. Bioluminescent image analysis was conducted using Living Image software (Perkin Elmer/Caliper Life Sciences, Hopkinton, MA, USA), and luciferase expression was reported as relative light units (photons/sec/cm^2^/sr).

### 4.17. Statistical Analysis

Continuous variables were tested for normal distribution using the Shapiro-Wilk normality test. Statistical analysis of normally distributed data was performed using an unpaired *t*-test or one-way analysis of variance (ANOVA) followed by Dunn’s multiple comparisons post-hoc test. The Mann-Whitney U-test was used to compare two groups of non-normally distributed data. For the animal studies, statistical analysis was performed using two-way ANOVA followed by Tukey’s multiple comparisons post-hoc test (normally distributed data). *p*-values of 0.05, 0.01, or 0.0001 were considered statistically significant. All calculations were carried out using PRISM 7.0 for Windows. 

## 5. Conclusions

In conclusion, instability of p53 in RCC is not associated with mutation of p53, but rather with increased expression of TGase 2 (because the p53 mutation rate in RCC is below 4%) [5]. Furthermore, our complex model showed that streptonigrin binds to unfolded TGase 2 at the β-sandwich domain of TGase 2. This binding could either affect the conformation of TGase 2 dimerization as an active cross-linking enzyme, see Figure S2, or interrupt binding of p53 to TGase 2, see Figure 2F. Finally, we used a xenograft model to demonstrate the potential therapeutic effects of streptonigrin against TGase 2 in RCC.

## Supplementary Materials

The following are available online at http://www.mdpi.com/2072-6694/10/11/455/s1.
