# Supplementary material for: Renal Cell Carcinoma Is Abrogated by p53 Stabilization through Transglutaminase 2 Inhibition
Source: Cancers (Basel). 2018 Nov 19;10(11):455. doi: 10.3390/cancers10110455 (PMC6267221; doi:10.3390/cancers10110455)
Supplement: Supplementary file 1 [file cancers-10-00455-s001.pdf]

Supplemental Materials: Renal Cell Carcinoma is Abrogated by p53 Stabilization through Transglutaminase 2 Inhibition

Seon-Hyeong Lee, Won-Kyu Lee, Nayeon Kim, Joon Hee Kang, Kyung-Hee Kim, Seul-Gi Kim, Jae-Seon Lee, Soohyun Lee, Jongkook Lee, Jungnam Joo, Woo Sun Kwon, Sun Young Rha, and Soo-Youl Kim

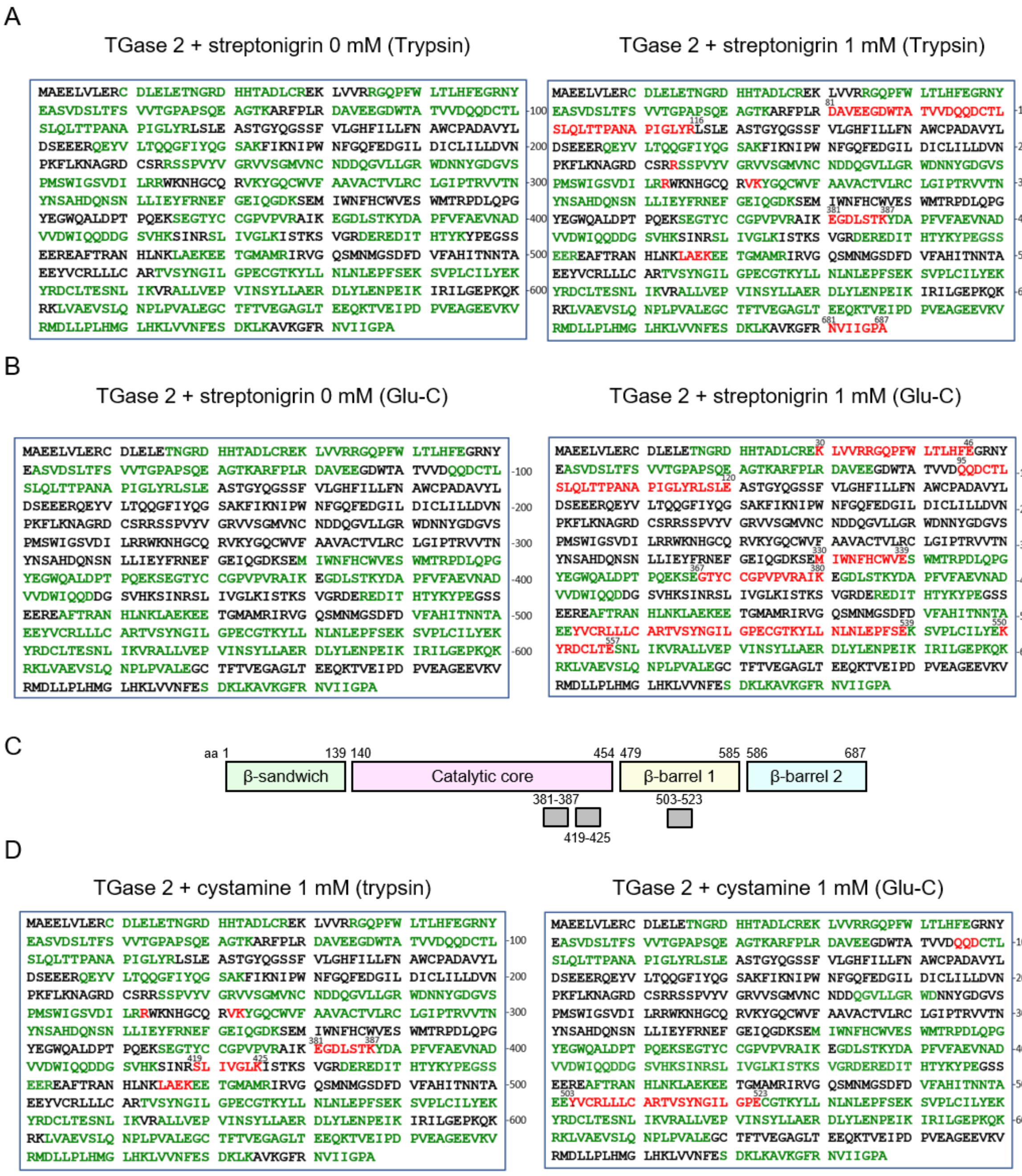
A
TGase 2 + streptonigrin 0 mM (Trypsin)
TGase 2 + streptonigrin 1 mM (Trypsin)
B
TGase 2 + streptonigrin 0 mM (Glu-C)
TGase 2 + streptonigrin 1 mM (Glu-C)
C
aa 1
139 140
454 479
585 586
687
β-sandwich
Catalytic core
β-barrel 1
β-barrel 2
381-387
419-425
503-523
D
TGase 2 + cystamine 1 mM (trypsin)
TGase 2 + cystamine 1 mM (Glu-C)

E

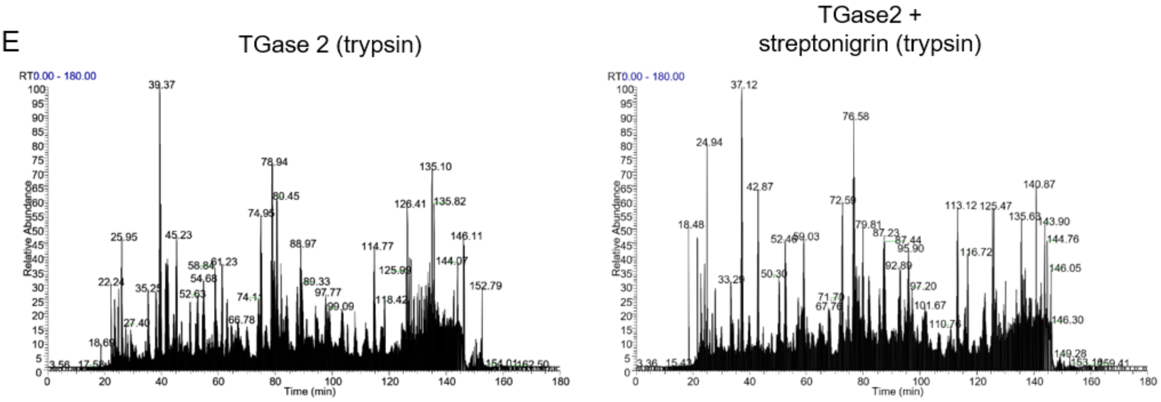

F

DAVEEGDWTATVVDQQDCTLSLQLTTPANAPIGLYR sequence spectrum (Trypsin)

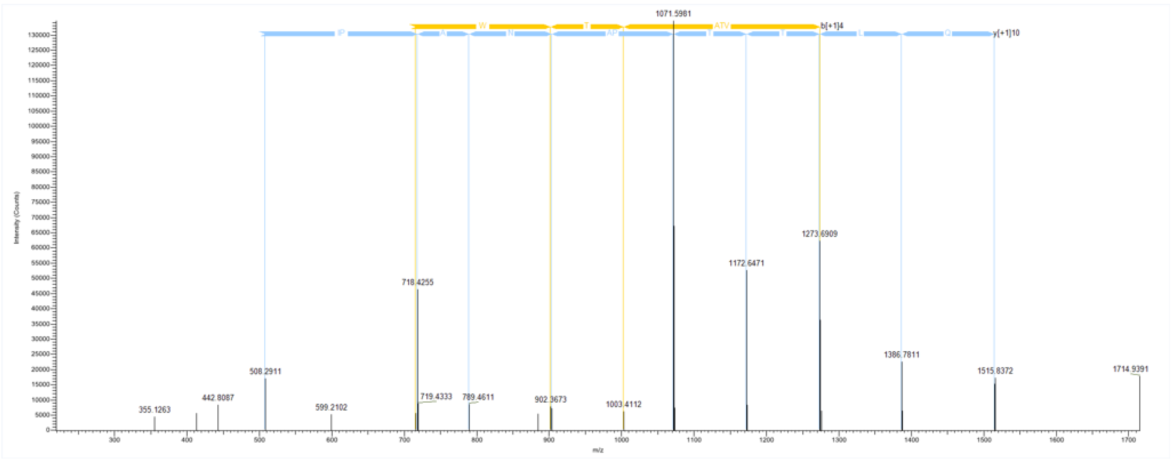

	(NH3+) - D>	A>	V>	E>	E>	G>	D>	W>	T>	A>	T>	V>	V>	D>	Q>	Q>	D>	C>
b (+1)	116.03423	187.07135	286.13977	415.18237	544.22497	601.24644	716.27339	902.35271	1003.40039	1074.43751	1175.48519	1274.55361	1373.62203	1488.64898	1616.70756	1744.76614	1859.79309	2019.82374
b*(+1)	99.00768	170.0448	269.11322	398.15582	527.19842	584.21989	699.24684	885.32616	986.37384	1057.41096	1158.45864	1257.52706	1356.59548	1471.62243	1599.68101	1727.73959	1842.76654	2002.79719
b°(+1)	98.02366	169.06078	268.1292	397.1718	526.2144	583.23587	698.26282	884.34214	985.38982	1056.42694	1157.47462	1256.54304	1355.61146	1470.63841	1598.69699	1726.75557	1841.78252	2001.81318
y (+1)	3832.86998	3761.83286	3662.76444	3533.72184	3404.67924	3347.65777	3232.63082	3046.5515	2945.50382	2874.4667	2773.41902	2674.3506	2575.28218	2460.25523	2332.19665	2204.13807	2089.11112	1929.08046
y*(+1)	3815.84343	3744.80631	3645.73789	3516.69529	3387.65269	3330.63122	3215.60427	3029.52495	2928.47727	2857.44015	2756.39247	2657.32405	2558.25563	2443.22868	2315.1701	2187.11152	2072.08457	1912.05391
y°(+1)	3814.85941	3743.82229	3644.75387	3515.71127	3386.66867	3329.6472	3214.62025	3028.54093	2927.49325	2856.45613	2755.40845	2656.34003	2557.27161	2442.24466	2314.18608	2186.1275	2071.10055	1911.0699

	T>	L>	S>	L>	Q>	L>	T>	T>	P>	A>	N>	A>	P>	I>	G>	L>	Y>	R - (COOH)
b (+1)	2120.87142	2233.95549	2320.98752	2434.07159	2562.13017	2675.21424	2776.26192	2877.3096	2974.36237	3045.39949	3159.44242	3230.47954	3327.53231	3440.61638	3497.63785	3610.72192	3773.78524	3929.88636
b*(+1)	2103.84487	2216.92894	2303.96097	2417.04504	2545.10362	2658.18769	2759.23537	2860.28305	2957.33582	3028.37294	3142.41587	3213.45299	3310.50576	3423.58983	3480.6113	3593.69537	3756.75869	3912.85981
b°(+1)	2102.86086	2215.94493	2302.97696	2416.06103	2544.11961	2657.20368	2758.25136	2859.29904	2956.35181	3027.38893	3141.43186	3212.46898	3309.52175	3422.60582	3479.62729	3592.71136	3755.77468	3911.8758
y (+1)	1828.03278	1714.94871	1627.91668	1514.83261	1386.77403	1273.68996	1172.64228	1071.5946	974.54183	903.50471	789.46178	718.42466	621.37189	508.28782	451.26635	338.18228	175.11896	3947.89693
y*(+1)	1811.00623	1697.92216	1610.89013	1497.80606	1369.74748	1256.66341	1155.61573	1054.56805	957.51528	886.47816	772.43523	701.39811	604.34534	491.26127	434.2398	321.15573	158.09241	3930.87038
y°(+1)	1810.02222	1696.93815	1609.90612	1496.82205	1368.76347	1255.6794	1154.63172	1053.58404	956.53127	885.49415	771.45122	700.4141	603.36133	490.27726	433.25579	320.17172	157.1084	3929.88636

G

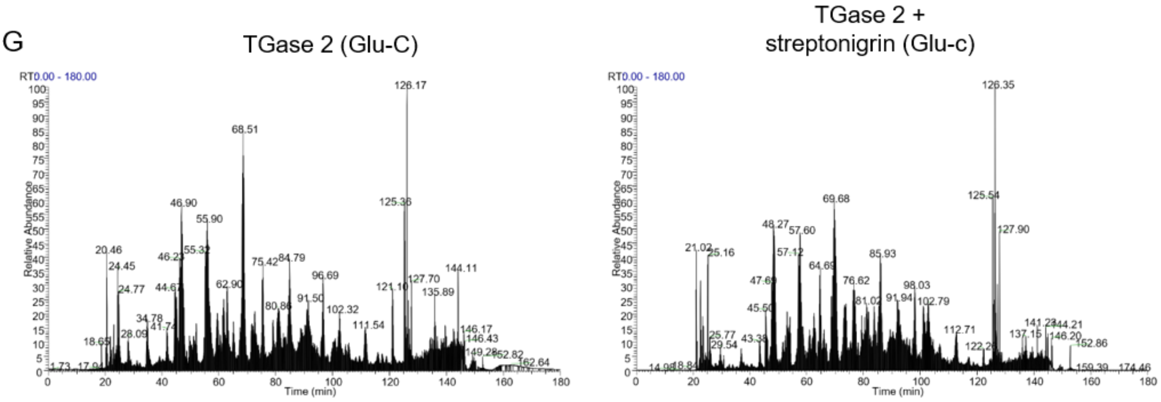

H

QQDCTLSLQLTTPANAPIGLYRLSLE sequence spectrum (Glu-C)

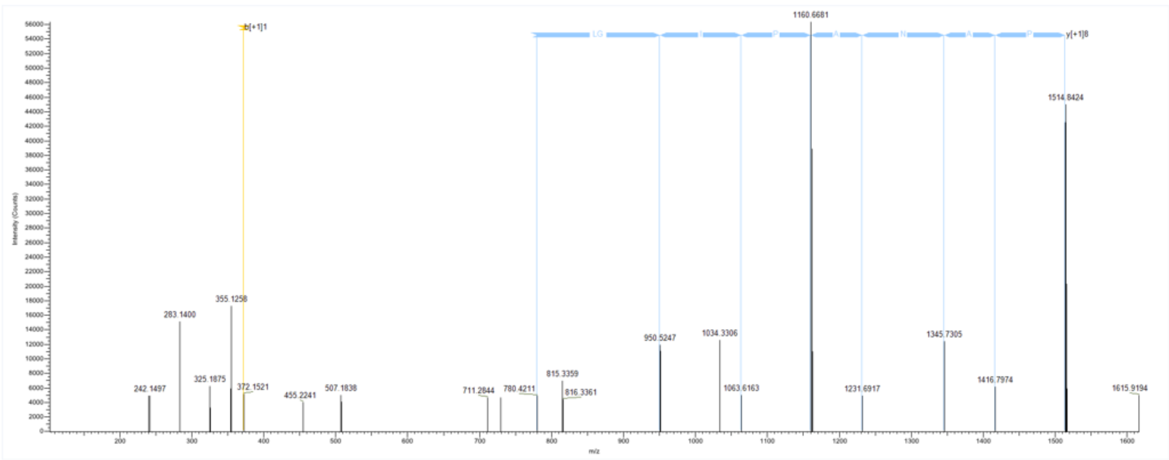

	(NH3+) - Q>	Q>	D>	C>	T>	L>	S>	L>	Q>	L>	T>	T>	P>
b (+1)	129.06586	257.12444	372.15139	532.18204	633.22972	746.31379	833.34582	946.42989	1074.48847	1187.57254	1288.62022	1389.6679	1486.72067
b*(+1)	112.03931	240.09789	355.12484	515.15549	616.20317	729.28724	816.31927	929.40334	1057.46192	1170.54599	1271.59367	1372.64135	1469.69412
b°(+1)	111.05529	239.11387	354.14082	514.17148	615.21916	728.30323	815.33526	928.41933	1056.47791	1169.56198	1270.60966	1371.65734	1468.71011
y (+1)	2774.43942	2646.38084	2531.35389	2371.32323	2270.27555	2157.19148	2070.15945	1957.07538	1829.0168	1715.93273	1614.88505	1513.83737	1416.7846
y*(+1)	2757.41287	2629.35429	2514.32734	2354.29668	2253.249	2140.16493	2053.1329	1940.04883	1811.99025	1698.90618	1597.8585	1496.81082	1399.75805
y°(+1)	2756.42885	2628.37027	2513.34332	2353.31267	2252.26499	2139.18092	2052.14889	1939.06482	1811.00624	1697.92217	1596.87449	1495.82681	1398.77404

	A>	N>	A>	P>	I>	G>	L>	Y>	R>	L>	S>	L>	E - (COOH)
b (+1)	1557.75779	1671.80072	1742.83784	1839.89061	1952.97468	2009.99615	2123.08022	2286.14354	2442.24466	2555.32873	2642.36076	2755.44483	2884.48743
b*(+1)	1540.73124	1654.77417	1725.81129	1822.86406	1935.94813	1992.9696	2106.05367	2269.11699	2425.21811	2538.30218	2625.33421	2738.41828	2867.46088
b°(+1)	1539.74723	1653.79016	1724.82728	1821.88005	1934.96412	1991.98559	2105.06966	2268.13298	2424.2341	2537.31817	2624.3502	2737.43427	2866.47687
y (+1)	1345.74748	1231.70455	1160.66743	1063.61466	950.53059	893.50912	780.42505	617.36173	461.26061	348.17654	261.14451	148.06044	2902.498
y*(+1)	1328.72093	1214.678	1143.64088	1046.58811	933.50404	876.48257	763.3985	600.33518	444.23406	331.14999	244.11796	131.03389	2885.47145
y°(+1)	1327.73692	1213.69399	1142.65687	1045.6041	932.52003	875.49856	762.41449	599.35117	443.25005	330.16598	243.13395	130.04988	2884.48743

Figure S1. Identification of streptonigrin binding site in TGase 2 by mass spectrometry analysis. Peptides produced by **(A)** trypsin and **(B)** Glu-C proteolytic digestion enzymes of the polymeric protein were unambiguously identified as streptonigrin binding sites by comparing the results of MALDI-TOF with the theoretical peptide masses. Identified sequences are marked in green (high confidence) while unidentified regions are marked in red. Sequence (95–116) is a putative streptonigrin binding region in merged peptides produced trypsin and glu-c. **(C–D)** To identify the binding site of cystamine on TGase 2, mass spectrometry analysis was employed following incubation of TGase 2 and cystamine for 30 min at RT. The mass coverage from samples revealed the difference between TGase 2 alone and the combination of TGase 2 and cystamine. The cystamine binding-mediated masking region of TGase 2 is denoted by underline 381–387 and 419–425 in solution trypsin digestion and 503–523 in solution Glu-C digestion. **(E)** Mass spectrum of TGase 2 only and TGase 2 and+ streptonigrin. MS analysis was employed to identify the streptonigrin binding site in TGase 2, following incubation of TGase 2 and

streptonigrin for 30 min at RT. Peptides produced by trypsin proteolytic enzyme of the polymeric enzyme. **(F)** The peptide consensus view for the 1316.64319 m/z. The sequence of the covered mass DAVEEGDWTATVVDQQDCTLSLQLTTPANAPIGLYR is shown. (**G**) Mass spectrum of TGase 2 only and TGase 2 + streptonigrin. MS analysis was employed to identify the streptonigrin binding site in TGase 2, following incubation of TGase 2 and streptonigrin for 30 min at RT. Peptides produced by Glu-C proteolytic enzyme of the polymeric enzyme. (**H**) The peptide consensus view for the 968.17450 m/z. The sequence of the covered mass QQDCTLSLQLTTPANAPIGLYRLSLE is shown.

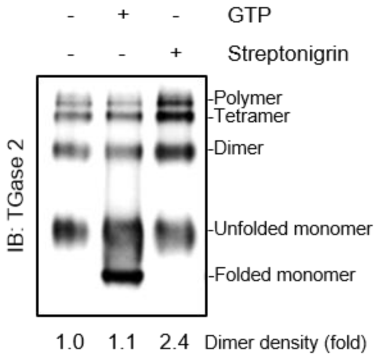

Figure S2. Analysis of TGase 2 conformation with GTP or streptonigrin by 10% native PAGE. TGase 2 was detected by western blotting using anti TGase 2 antibody after incubation of TGase 2 with 1 mM of GTP or 1 μM of streptinigrin for 30 min at 37°C. Lane 1: TGase 2 was incubated without GTP. Lane 2: incubation of TGase 2 with GTP. Lane 3: incubation of TGase 2 with streptonigrin.

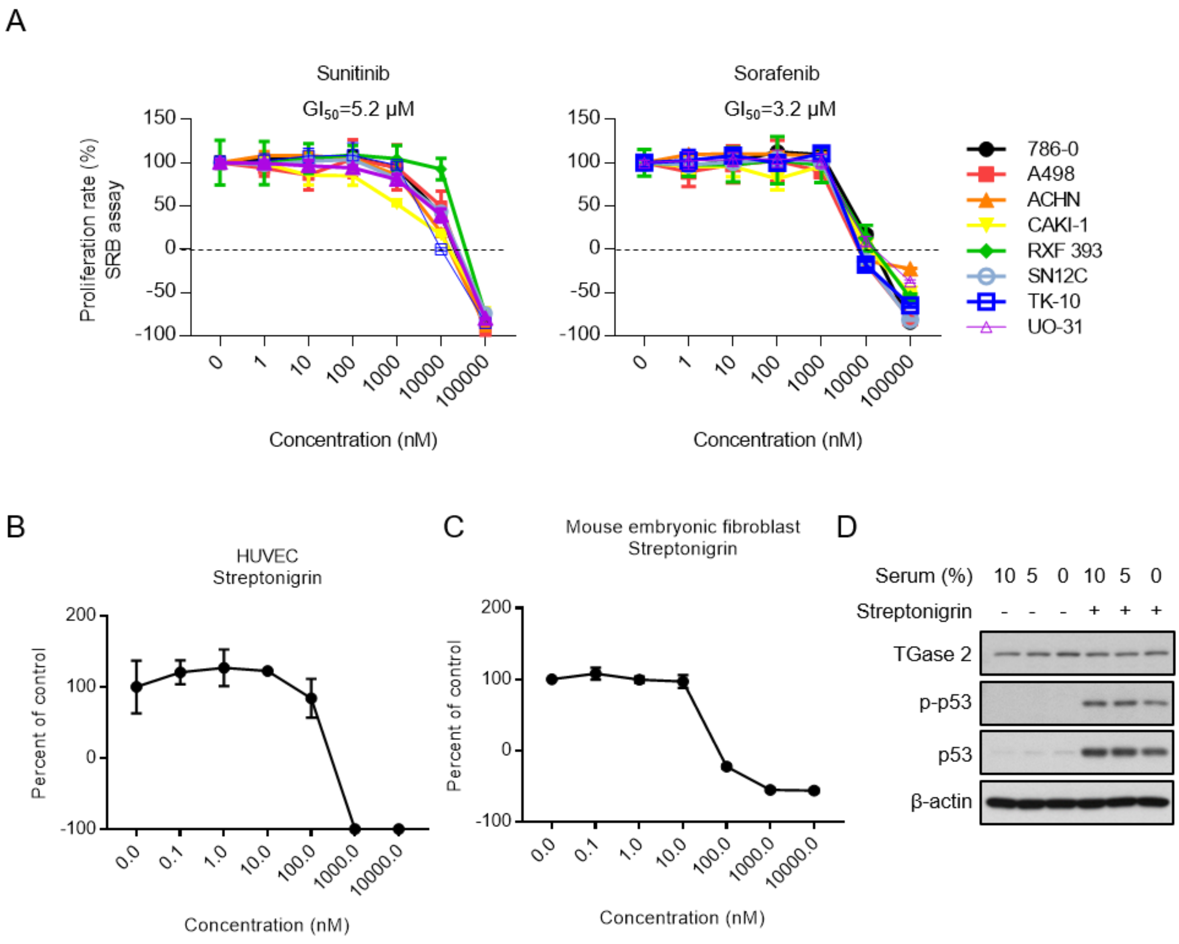

Figure S3. Anti-proliferative activities of streptonigrin, sunitinib, and sorafenib were evaluated by the sulforhodamin B (SRB) assay. (**A**–**B**) SRB assay of sunitinib and sorafenib cytotoxicity in a panel of human RCC cells. (**A**) Sunitinib (GI50: 5.2 μM) and sorafenib (GI50: 3.2 μM) were measured. (**B**) SRB assay of streptonigrin (GI_{50}: 117.5 nM) in human umbilical vein endothelial cells (HUVEC). (**C**) SRB assay of streptonigrin (GI_{50}: 24.7 nM) in mouse embryonic fibroblast (MEF) wild-type cells. (**D**) CAKI-1 cells were incubated with 10%, 5%, or 0 % serum concentration for 24 h and treated with streptonigrin (0 or 100 nM) for 4 h.

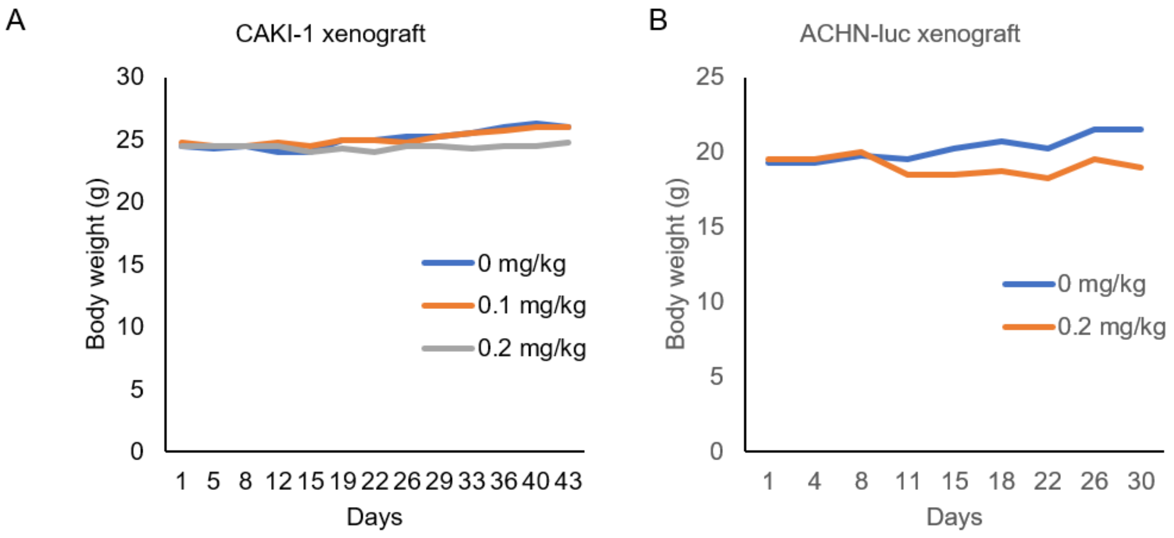

Figure S4. Mouse body weight measured during drug treatment. (**A**–**B**) The body weight did not change during the streptonigrin treatments in CAKI-1 and ACHN-luc mouse xenograft models from Figure 4.

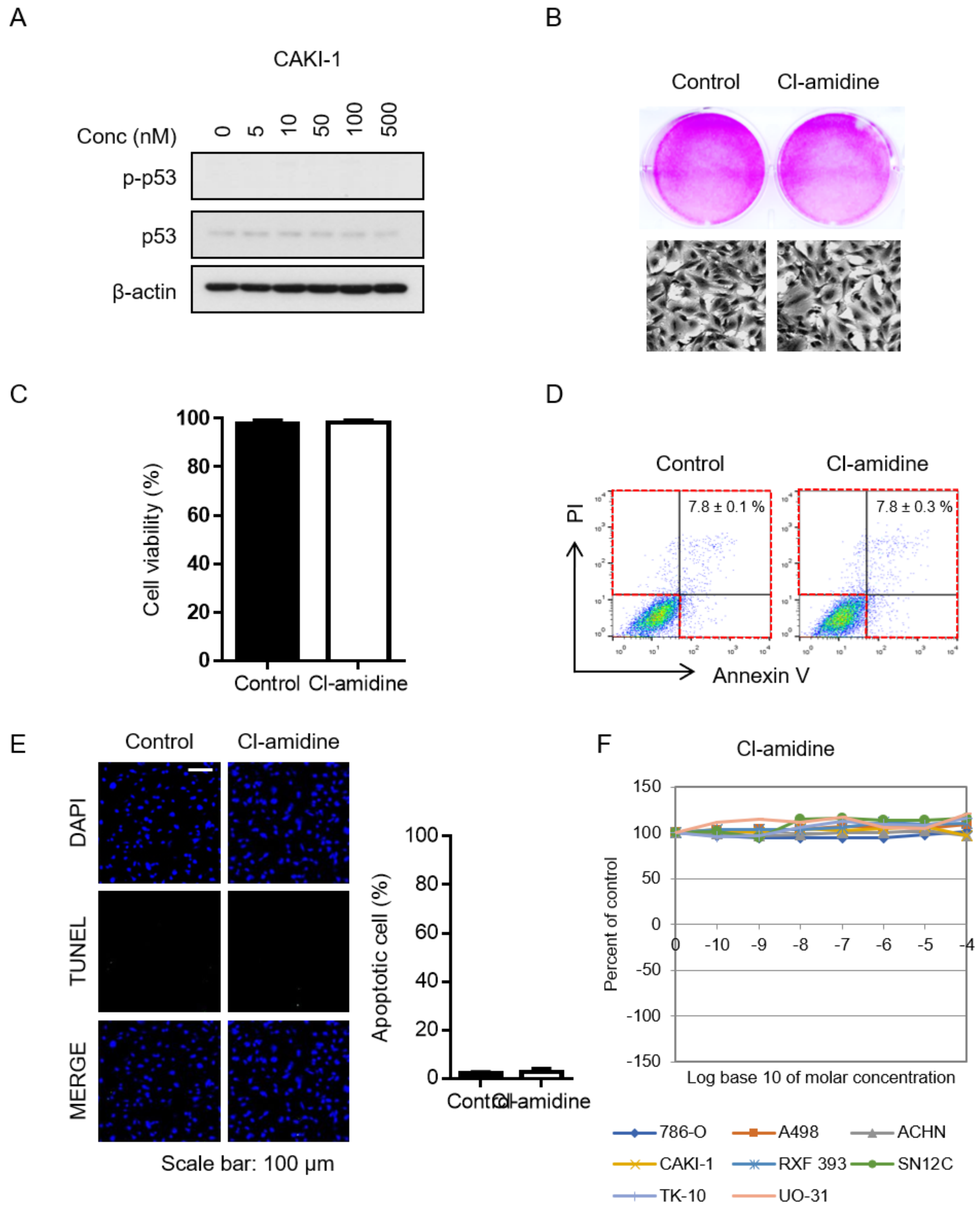

Figure S5. PAD inhibitor does not induce apoptosis in CAKI-1 cells. (**A**) Cl-amidine (PAD inhibitor) not rescued p-p53 and p53 in a dose-dependent manner. (**B**) Cl-amidine not suppressed cell proliferation in cells. After treatment with Cl-amidine (0 or 500 nM) for 6 h, the cells were fixed in 4 % paraformaldehyde and stained with sulforhodamin B (original magnification 10×). (**C**) Cell viability was determined by trypan blue staining assay treated Cl-amidine (0 or 500 nM) in cells. (**D**) After treatment with Cl-amidine (0 or 500 nM) for 6 h in cells, it was analyzed by FACS after staining with Annexin V/propidium iodside. (**E**) Cells were exposed to Cl-amidine (0 or 500 nM) for 6 h and apoptosis was analyzed by TUNEL assay. A bar graph shows the percentage (mean) of apoptotic cells in at least four randomly selected fields of

view. Scale bar = 100 µm. (**F**) Results of sulforhodamin B assay of Cl-amidine cytotoxicity in human RCC cells exposed to the indicated concentrations. The cells were incubated with compounds for 48 h. Data represent three independent experiments.

Table S1. Short tandem repeat (STR) profiling of cell lines. The 10 ng of genomic DNA was amplified with an amplification PCR kit *GenePrint*® 10 System (Promega, Madison, WI, USA), which is a multiplex STR test that co-amplifies in a single polymerase chain reaction (PCR) 10 loci: TH01, D21S11, D5S818, D13S317, D7S820, D16S539, CSF1PO, Amelogenin, vWA, TPOX). Amplification was performed following suggested instructions, using the thermal cycler GeneAmp PCR system 9700 (Thermo Fisher Scientific, Waltham, MA, USA). To control the PCR amplification process, each reaction contains negative and positive controls. Amplifed DNA 1 ul was mixed with 9.5 ul of Hi-Di formamide (Thermo Fisher Scientific, Waltham, MA, USA) and 0.5 ul of ILS600 (kit supplied size marker). After denaturation at 95°C for 3min, the final sample was loaded into the ABI3730 sequencer (Applied Biosystems Life Technologies, Foster City, CA, USA) with POP7 polymer in a 50-cm capillary. After electrophoresis, the STR profile was analyzed using GeneMapper v 5.0 software.

STR Profile

Sample		TH01	D21S11	D5S818	D13S317	D7S820	D16S539	CSFIPO	AMEL	vWA	TPOX
786-O	Reference	6, 9.3	29, 30	9	8	11, 12	12	10	X, Y	15, 17	8, 11
	Result	6, 9.3	29, 30	9	8	11, 12	12	10	X, Y	15, 17	8, 11
A498	Reference	6, 9.3	28, 32	11, 13	12	10, 11	12	11, 12	X	18	8, 11
	Result	6, 9.3	28, 32	11, 13	12	10, 11	12	11, 12	X	18	8, 11
ACHN	Reference	8	30	12	12	9, 11	12, 13	11	X	16,17	8, 11
	Result	8	30	12	12	9, 11	12, 13	11	X	16,17	8, 11
ACHN-luc	Result	8	30	12	12	9, 11	12, 13	11	X	16,17	8, 11
CAKI-1	Reference	6, 8	28, 30	11, 12	11, 12	12	12	10, 11	X	15, 17	8, 11
	Result	6, 8	28, 30	11, 12	11, 12	12	12	10, 11	X	15, 17	8, 11
RXF-393	Reference	7, 8	28, 33.2	12,13	9, 12	11, 12	11	10, 12	X	16, 17	8, 11
	Result	7, 8	28, 33.2	12,13	9, 12	11, 12	11	10, 12	X	16, 17	8, 11
SN12c	Reference	6, 8	29, 30	11	9	9	11	9, 10	X	15	8, 11
	Result	6, 8	29, 30	11	9	9	11	9, 10	X	15	8, 11
TK-10	Reference	8	29	11, 12	9	10, 11	12	12	X	16, 20	11
	Result	8	29	11, 12	9	10, 11	12	12	X	16, 20	11
UO-31	Reference	7	32.2	11, 12	9, 11	10	11, 13	10, 12	X	16, 20	11
	Result	7	32.2	11, 12	9, 11	10	11, 13	10, 12	X	16, 20	11
HCT116	Reference	8, 9	29, 30	10, 11	10, 12	11, 12	11, 13	7, 10	X	17, 21	8
	Result	8, 9	29, 30	10, 11	10, 12	11, 12	11, 3	7, 10	X	17, 21, 22	8, 9
HEK293	Reference	7, 9.3	28, 30.2	8, 9	12, 14	11, 12	9, 13	11, 12	X	16, 19	11
	Result	7, 9.3	28, 30.2	8, 9	12, 14	11, 12	9, 13	11, 12	X	16, 19	11

Table S2. Primers used in this paper.

Application	Name	Sequence (5'–3')	vector
Primers for mutagenesis	TGase 2 mutant_antisense	gttggccggggtggtgagcGCcagcgagagggtgcagtctGCcGCgtccaccacggtggctg	HA-pcDNA3.1
	TGase 2 mutant_sense	ctcaccaccccggccaacgcccccatcggcctgtatGCcctcagcctggaggcctc	
	insert_1	ctagacatctgcctgatc	
	insert_2	tttgcggaggtcaatgcc	
Primers for cloning	p53_Forward	tgacgatgacaagcttatggaggagccgcagtcag	p3xFlag-CMV
	p53_Reverse	tcgcggccgcaagctttcagtctgagtcaggcccttc	

Table S3. Comparative GI_{50}, TGI, and LC_{50} of streptonigrin, sutent, and sorafenib. Growth Inhibition of 50% (GI_{50}), total growth inhibition (TGI), and lethal concentration of 50% (LC_{50}) of different drugs are calculated in RCC cells after 48 h treatment.

GI_{50} Value - log10(M)

Cell Name	Chemical Name		
	Streptonigrin	Sutent	Sorafenib
786-O	-8.2	-5.2	-5.4
A498	-8.0	-5.0	-5.7
ACHN	-8.4	-5.5	-5.5
CAKI-1	-9.1	-5.9	-5.5
RXF 393	-7.2	-4.7	-5.4
SN12c	-7.7	-5.2	-5.6
TK-10	-8.1	-5.5	-5.5
UO-31	-7.5	-5.3	-5.4
Average Value	-8.0<br>9.4 nM	-5.3<br>5.2 μM	-5.5<br>3.2 μM

TGI Value - log10(M)

Cell Name	Chemical Name		
	Streptonigrin	Sutent	Sorafenib
786-O	-7.5	-4.7	-4.8
A498	-7.3	-4.7	-5.2
ACHN	-6.9	-4.8	-5.1
CAKI-1	-7.5	-4.8	-4.9
RXF 393	-6.3	-4.5	-4.8
SN12c	-7.1	-4.6	-5.1
TK-10	-7.3	-5.0	-5.1
UO-31	-7.0	-4.7	-4.8
Average Value	-7.1<br>77.1 nM	-4.7<br>18.8 μM	-5.0<br>10.6 μM

LC_{50} Value - log10(M)

Cell Name	Chemical Name		
	Streptonigrin	Sutent	Sorafenib
786-O	-6.1	-4.3	-4.3
A498	-5.6	-4.3	-4.5
ACHN	-5.3	-4.4	-
CAKI-1	-4.8	-4.2	-4.0
RXF 393	-5.0	-4.2	-4.1
SN12c	-5.3	-4.2	-4.5
TK-10	-5.7	-4.4	-4.3
UO-31	-4.8	-4.3	-
Average Value	-5.3<br>4.7 μM	-4.3<br>51.5 μM	-4.3<br>52.1 μM
